# Handover of anesthesia care is associated with an increased risk of delirium in elderly after major noncardiac surgery: results of a secondary analysis

**DOI:** 10.1007/s00540-019-02627-3

**Published:** 2019-02-28

**Authors:** Guang-Yu Liu, Xian Su, Zhao-Ting Meng, Fan Cui, Hong-Liang Li, Sai-Nan Zhu, Dong-Xin Wang

**Affiliations:** 1 Department of Anesthesiology and Critical Care Medicine, Peking University First Hospital, No. 8 Xishiku Street, Beijing, 100034 China; 2 Department of Critical Care Medicine, Peking University Third Hospital, Beijing, China; 3 Department of Biostatistics, Peking University First Hospital, Beijing, China

**Keywords:** Elderly, Noncardiac surgery, Handover, Postoperative outcome, Delirium

## Abstract

In patients undergoing major surgery, complete handover of intraoperative anesthesia care is associated with adverse postoperative outcomes including high mortality and more major complications. The purpose of this study was to explore the association between the intraoperative complete handover between anesthesiologists and the occurrence of postoperative delirium. This was a secondary analysis of the database of a previously published clinical trial. Seven hundred patients aged 65 years or older, who were admitted to the intensive care unit after noncardiac surgery, were included. Delirium was assessed with the Confusion Assessment Method for the Intensive Care Unit twice daily during the first 7 postoperative days. Other postoperative outcomes were also monitored. The association between the intraoperative complete handover of anesthesia care and the development of postoperative delirium was analyzed with a logistic regression model. Of the 700 enrolled patients, 111 (15.9%) developed postoperative delirium within 7 days. After correction for confounding factors, intraoperative complete handover between anesthesiologists was associated with an increased risk of postoperative delirium (OR 1.787, 95% CI 1.012–3.155, *P* = 0.046). Patients with intraoperative complete handover also had higher incidence of non-delirium complications (*P* = 0.003) and stayed longer in hospital after surgery (*P* = 0.002). For elderly patients admitted to the intensive care unit after noncardiac surgery, intraoperative complete handover of anesthesia care was associated with an increased risk of postoperative delirium. Chinese Clinical Trial Registry (http://www.chictr.org.cn): ChiCTR-TRC-10000802.

**Electronic supplementary material:**

The online version of this article (10.1007/s00540-019-02627-3) contains supplementary material, which is available to authorized users.

## Introduction

Delirium is an acutely occurring cerebral dysfunction characterized with transient and fluctuating disturbances in attention, consciousness and cognition. It is a common complication in the elderly after surgery, with reported incidence varying from 3.6 to 54.4% [1–3]. Delirium development is associated with worse outcomes, including prolonged hospital stay, increased medical costs, elevated readmission rates, declined quality of life, and shortened long-term survival [4–6]. The occurrence of postoperative delirium is a result of the interaction of multiple factors, including predisposing and precipitating factors [4, 6–8]. Being greater than 65 years old and admission to intensive care unit (ICU) are significant risk factors, and patients with these characteristics may have a delirium incidence up to 87% [9].

With the aging population and the increasing number of surgical cases [10, 11], intraoperative handover of anesthesia care is inevitable in some cases due to personal problem, such as fatigue or illness, or department commitments [12]. Handover can be temporary (initial anesthesiologist returns after a break) or complete (initial anesthesiologist no longer returns) [13]. After complete handovers, the incoming anesthesiologist receives all the information in a busy environment with many distractions. Studies showed that a high proportion of intraoperative handover between anesthesiologists is insufficient [14], and that complete handover of anesthesia care is associated with worse outcomes, including increased all-cause death and major complications within 30 days after surgery [13]. However, the impact of anesthesia handover on postoperative delirium remains unclear.

The purpose of this secondary analysis was to analyze the association between complete handover of anesthesia care and the risk of delirium development in elderly patients who were admitted to ICU after noncardiac surgery.

## Methods

### Study design

This was a secondary analysis of the database of a previously published clinical trial [15]. The purpose of the original trial was to explore the role of dexmedetomidine in preventing postoperative delirium in critically ill elderly patients. The study protocol was approved by the Clinical Research Ethics Committee of Peking University First Hospital (number 2011 [10]) and registered with Chinese Clinical Trial Registry (http://www.chictr.org.cn, number ChiCTR-TRC-10000802). The original study was conducted from August 17, 2011 to November 20, 2013 in the ICUs of Peking University First Hospital and Peking University Third Hospital. Written informed consents were obtained from patients, their next of kin or their legal representatives. Approval from the ethics committee for this secondary analysis was waived because the analysis was based on completely anonymized data without personal identification code. The privacy of participants was strictly observed.

### Patients

The inclusion criteria were elderly patients (age ≥ 65 years) who were admitted to the ICU after elective noncardiac surgery under general anesthesia. Patients who met any of the following criteria were excluded: (1) preoperative history of schizophrenia, epilepsy, Parkinson’s disease, or myasthenia gravis; (2) inability to communicate because of coma, severe dementia or language barriers before surgery; (3) brain trauma or neurosurgery; (4) preoperative left ventricular ejection fraction (LVEF) < 30%, sick sinus syndrome, heart rate < 50 beats/min or two degree or higher atrioventricular block without pacemaker; (5) severe liver dysfunction (Child-Pugh C grade) or severe renal dysfunction (preoperative renal replacement therapy); or (6) expected survival ≤ 24 h.

### Handover of patient care

During surgery, complete handover of anesthesia care was defined as transfer of patient care from one anesthesiologist to another and the original anesthesiologist did not return [13]. In the participating hospitals, handover of anesthesia care occurred between 4 and 5 pm and was marked in the electronically recorded worksheet. At the end of surgery, all patients were transferred to the ICU, and handover of patient care occurred between the anesthesiologists and the ICU physicians.

### Delirium assessment

Delirium was assessed twice daily (8–10 am and 6–8 pm) during the first 7 days after surgery. This was performed with the Confusion Assessment Method for the Intensive Care Unit (CAM-ICU) in two steps [16]. First, the state of sedation/agitation was assessed using the Richmond Agitation Sedation Scale (RASS) [17]. For patients who were intubated and mechanically ventilated, sedative (propofol and/or midazolam) administration was titrated to a RASS of − 2 or higher until extubation. For those who were deeply sedated or unarousable (RASS − 4 to − 5), delirium was not assessed and patients were recorded as comatose; for those with a RASS score of − 3 or higher, delirium was assessed using the CAM-ICU which detected four features, i.e., (1) an acute onset or fluctuation course; (2) inattention; (3) thinking disorder; and (4) altered level of consciousness. Delirium was diagnosed when patients presented features 1, 2, 3 or 1, 2, 4. Investigators who performed delirium assessment were trained by a psychiatrist to use the CAM-ICU before the study period.

### Data collection

Demographic information included gender, age, body mass index (BMI), and years of education. Baseline data included admission diagnosis, preoperative comorbidities, previous history of surgery, preoperative laboratory test results, and the American Society of Anesthesiologists (ASA) classification. Intraoperative data included method and duration of anesthesia, use of anesthetics and analgesics, use of glucocorticoids, type and duration of surgery, estimated blood loss, as well as fluid infusion and blood transfusion. Grade of surgery is rated according to the *NICE Guidance of routine preoperative tests for elective surgery* [18]. Cardiac risk of surgery is rated according to the *ACC*/*AHA 2007 guidelines on perioperative cardiovascular evaluation and care for noncardiac surgery* [19]. Postoperative data included type of patient-controlled analgesia, supplemental sedatives and analgesics, prophylactic dexmedetomidine infusion (continuous infusion at a rate of 0.1 µg/kg/h from ICU admission to 8 am of next morning) [15], use and duration of mechanical ventilation, length of stay in ICU and hospital, occurrence of non-delirium complications within 30 days, and all-cause 30-day mortality. Non-delirium complications were generally defined as new-onset medical events other than delirium that were harmful to patients’ recovery and required therapeutic intervention (Online Resource 1). Investigators who performed patient recruitment and data collection were trained before the study period.

### Statistical analysis

Patients were divided into two groups according to the presence of complete anesthesia handover or not. Continuous variables were presented as mean ± SD and analyzed with independent samples *t* test or Mann–Whitney *U* test. Categorical variables were presented as number (%) and analyzed with the Chi square test or Fisher’s exact test. Time-to-event variables were presented as median (95% confidence interval of median) analyzed with the Kaplan–Meier estimator, with differences compared with the Log-rank test. Factors that might be associated with the development of postoperative delirium were screened with univariate logistic regression analyses, and those with a *P* value < 0.05 were included in a multivariate logistic regression model to assess the risk-adjusted association between the complete handover of anesthesia care and the development of postoperative delirium. SPSS 25.0 software package (SPSS Inc, Chicago, Ill) was used for statistical analysis. Two-sided *P* values of less than 0.05 were considered statistically significant.

## Results

### Patient recruitment

**Fig. 1:**
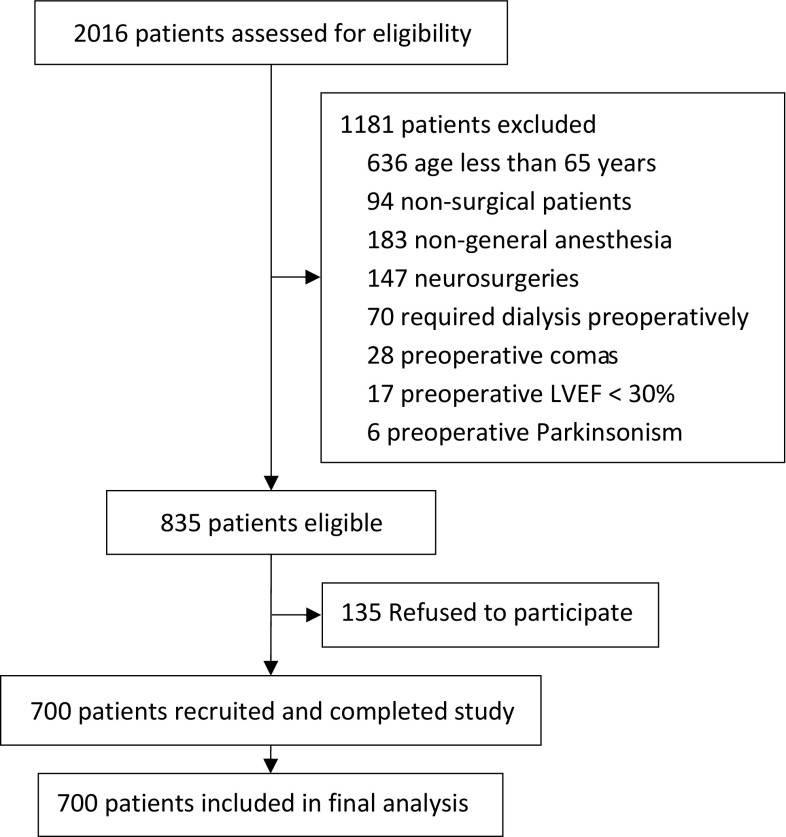
Flowchart of the study

From August 2011 to November 2013, 2016 patients were screened; of these, 835 were eligible, and 700 were enrolled and completed the study (Fig. 1). Compared with patients without completed handover of anesthesia care, those with complete handover were younger (*P* = 0.001), suffered less hypertension before surgery (*P* = 0.045), underwent longer anesthesia and surgery (all *P* < 0.001) and more major or complex surgery (*P* = 0.006), lost more blood but received more fluid infusion and blood transfusion during surgery (all *P* < 0.001), and received more mechanical ventilation as well as propofol sedation during ICU stay after surgery (all *P* < 0.001) (Tables 1, 2).

**Table 1:** Baseline variables

Variable	All patients (*n* = 700)	With complete handover (*n* = 102)	Without complete handover (*n* = 598)	*P* value
Age, years	74.3 ± 6.8	72.3 ± 6.7	74.7 ± 6.8	0.001
Male gender	423 (60.4)	67 (65.7)	356 (59.5)	0.240
Body mass index, kg/m^2^	23.7 ± 3.9	23.8 ± 4.2	23.7 ± 3.9	0.739
Education, years	9.0 (6.0, 12.0)	9.0 (6.0, 12.0)	9.0 (6.0, 12.0)	0.779
Preoperative comorbidity				
Previous stroke	161 (23.0)	21 (20.6)	140 (23.4)	0.531
Hypertension	446 (63.7)	56 (54.9)	390 (65.2)	0.045
Coronary heart disease	232 (33.1)	27 (26.5)	205 (34.3)	0.121
COPD	42 (6.0)	5 (4.9)	37 (6.2)	0.613
Chronic smoking^a^	176 (25.1)	32 (31.4)	144 (24.1)	0.117
Diabetes mellitus	190 (27.1)	21 (20.6)	169 (28.3)	0.107
Liver injury^b^	19 (2.7)	2 (2.0)	17 (2.8)	> 0.999
Renal injury^c^	35 (5.0)	4 (3.9)	31 (5.2)	0.589
Alcoholism^d^	63 (9.0)	11 (10.8)	52 (8.7)	0.496
Previous surgery	398 (56.9)	53 (52.0)	345 (57.7)	0.280
Chronic benzodiazepines	61 (8.7)	6 (5.9)	55 (9.2)	0.273
Preoperative laboratory tests				
Hematocrit < 30%	106 (15.1)	17 (16.7)	89 (14.9)	0.642
Albumin < 30 g/L	50 (7.1)	8 (7.8)	42 (7.0)	0.766
Glucose < 4.0 or > 10.0 mmol/L	57 (8.1)	4 (3.9)	53 (8.9)	0.092
Na^+^ < 135.0 or > 145.0 mmol/L	67 (9.6)	9 (8.8)	58 (9.7)	0.781
K^+^ < 3.5 or > 5.5 mmol/L	73 (10.4)	8 (7.8)	65 (10.9)	0.355
ASA physical status				0.130
Class II	398 (56.9)	65 (63.7)	333 (55.7)	
Class III	302 (43.1)	37 (36.3)	265 (44.3)	

Data are presented as mean ± SD, number (%), or median (interquartile range)

*COPD* chronic obstructive pulmonary disease, *ASA* American Society of Anesthesiologists

^a^Daily smoking of cigarettes up to half a pack for at least 2 years

^b^Alanine aminotransferase and/or aspartate aminotransferase higher than five times of the normal upper limit

^c^Serum creatinine level ≥ 177 µmol/L

^d^Two drinks or more daily, or weekly consumption of the equivalent of 150 mL of alcohol

**Table 2:** Perioperative variables

Variable	All patients (*n* = 700)	With complete handover (*n* = 102)	Without complete handover (*n* = 598)	*P* value
Benzodiazepines at preoperative night	79 (11.3)	12 (11.8)	67 (11.2)	0.869
Type of anesthesia				0.233
General	578 (82.6)	80 (78.4)	498 (83.3)	
Combined epidural-general	122 (17.4)	22 (21.6)	100 (16.7)	
Intraoperative medication				
Nitrous oxide	523 (74.7)	73 (71.6)	450 (75.3)	0.429
Sevoflurane	501 (71.6)	73 (71.6)	428 (71.6)	0.999
Benzodiazepines	326 (46.6)	51 (50.0)	275 (46.0)	0.453
Propofol	634 (90.6)	97 (95.1)	537 (89.8)	0.091
Etomidate	273 (39.0)	38 (37.3)	235 (39.3)	0.696
Glucocorticoids	646 (92.3)	98 (96.1)	548 (91.6)	0.120
Duration of anesthesia, min	288 (211, 386)	416 (274, 586)	274 (202, 362)	< 0.001
Duration of surgery, min	200 (126, 292)	312 (190, 479)	189 (119, 271)	< 0.001
Type of surgery				0.153
Superficial and transurethral	69 (9.9)	4 (3.9)	65 (10.9)	
Intra-abdominal	475 (67.9)	71 (69.6)	404 (67.6)	
Intra-thoracic	120 (17.1)	21 (20.6)	99 (16.5)	
Spinal and extremital	36 (5.1)	6 (5.9)	30 (5.0)	
Grade of surgery^a^				0.006
Intermediate	42 (6.0)	0 (0.0)	42 (7.0)	
Major or complex	658 (94.0)	102 (100.0)	556 (93.0)	
Cardiac risk of surgery^b^				0.281
Low	33 (4.7)	2 (2.0)	31 (5.2)	
Intermediate	658 (94.0)	98 (96.1)	560 (93.6)	
High	9 (1.3)	2 (2.0)	7 (1.2)	
Estimated blood loss, ml	150 (50, 450)	400 (100, 800)	100 (40, 400)	< 0.001
Total intraoperative fluid, ml	2510 (1600, 3700)	3600 (2488, 5313)	2350 (1600, 3413)	< 0.001
Intraoperative blood transfusion	114 (16.3)	30 (29.4)	84 (14.0)	< 0.001
ICU admission with intubation	382 (54.6)	82 (80.4)	300 (50.2)	< 0.001
Prophylactic dexmedetomidine	350 (50.0)	52 (51.0)	298 (49.8)	0.830
Postoperative analgesia				0.051
None	73 (10.4)	5 (4.9)	68 (11.4)	
PCIA	516 (73.7)	75 (73.5)	441 (73.7)	
PCEA	111 (15.9)	22 (21.6)	89 (14.9)	
Other sedatives/analgesics within 7 days				
Propofol	357 (51.0)	80 (78.4)	277 (46.3)	< 0.001
Benzodiazepines	58 (8.3)	11 (10.8)	47 (7.9)	0.322
Opioids	201 (28.7)	32 (31.4)	169 (28.3)	0.521
NSAIDs	229 (32.7)	31 (30.4)	198 (33.1)	0.589
Pathologically diagnosed cancer	561 (80.1)	84 (82.4)	477 (79.8)	0.545

Data are presented as number (%), or median (interquartile range)

*ICU* intensive care unit, *PCIA* patient-controlled intravenous analgesia, *PCEA* patient-controlled epidural analgesia, *NSAIDs* non-steroid anti-inflammatory drugs

^a^Rated according to *NICE Guidance of Routine preoperative tests for elective surgery* [18]

^b^Rated according to *ACC*/*AHA 2007 guidelines on perioperative cardiovascular evaluation and care for noncardiac surgery* [19]

### Unadjusted postoperative outcomes

**Fig. 2:**
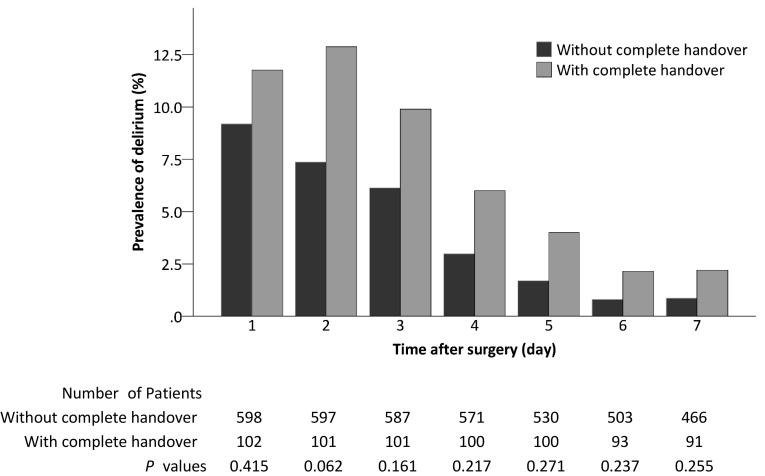
Daily prevalence of postoperative delirium in patients with or without complete handover of intraoperative anesthesia care. Some patients died or discharged from hospital within 7 days after surgery, leading to different sample sizes during this period

**Fig. 3:**
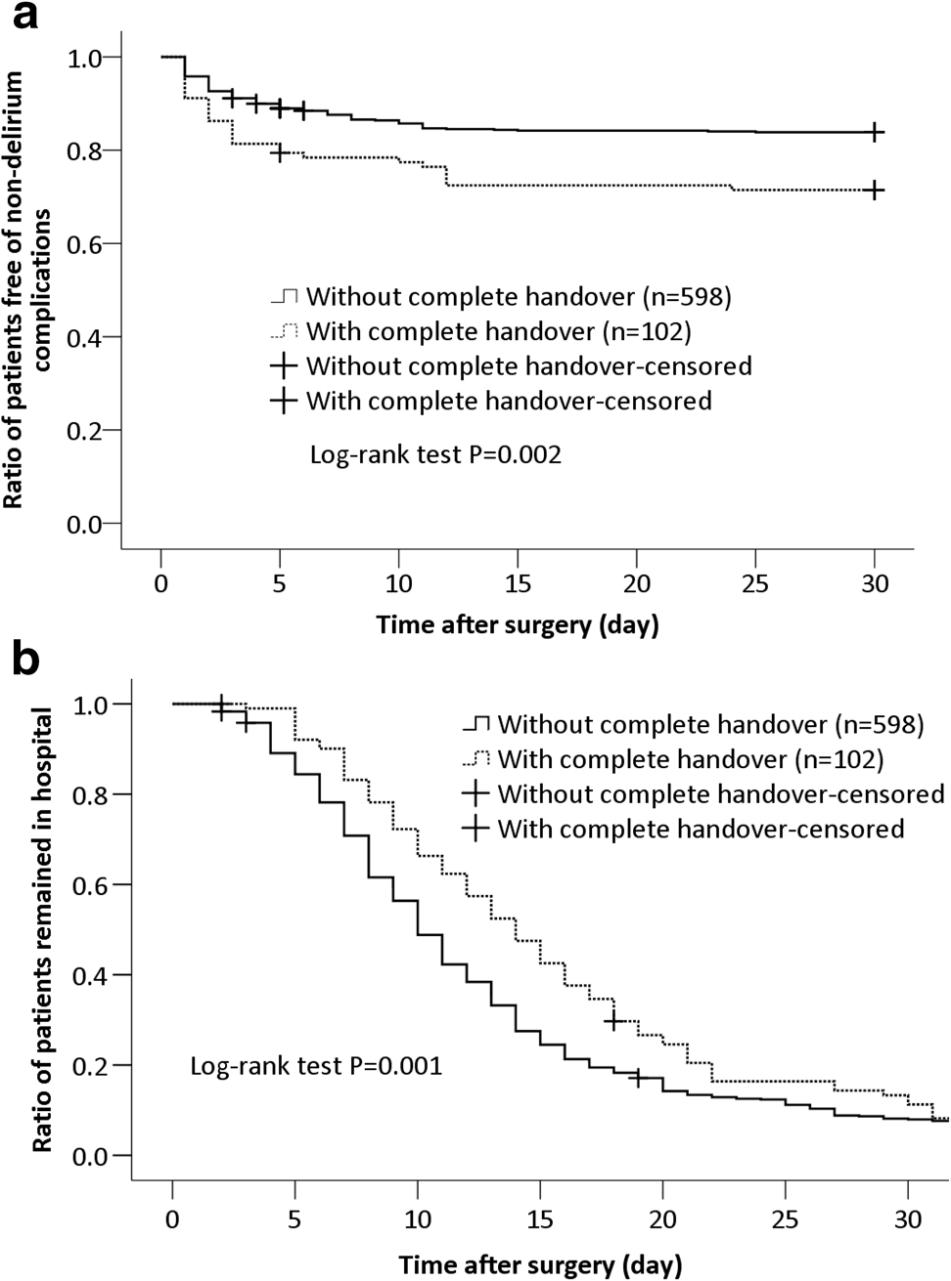
The occurrence of non-delirium complications (**a**) and the length of stay in hospital after surgery (**b**) in patients with or without complete handover of intraoperative anesthesia care

Compared with patients without complete handover of anesthesia care, those with complete handover had a higher incidence of delirium within 7 days after surgery [22.6% (23/102) vs. 14.7% (88/598), *P* = 0.045] (Fig. 2); they also had a higher incidence of non-delirium complications within 30 days [28.4% (29/102) vs. 16.0% (96/598), *P* = 0.003] and stayed longer in hospital after surgery [14.0 days (95% CI 12.0, 16.0) vs. 10.0 days (9.4, 10.6), *P* = 0.002] (Table 3; Fig. 3a, b).

**Table 3:** Postoperative outcomes

Variable	All patients (*n* = 700)	With complete handover (*n* = 102)	Without complete handover (*n* = 598)	*P* value
Delirium within 7 days	111 (15.9)	23 (22.5)	88 (14.7)	0.045
Length of stay in ICU, h	21.1 (20.6, 21.5)	17.7 (16.1, 19.3)	21.3 (20.9, 21.8)	0.336
Time to extubation, h	5.2 (4.2, 6.3) (*n* = 382)	8.7 (7.3, 10.0) (*n* = 82)	4.5 (3.7, 5.2) (*n* = 300)	0.054
Non-delirium complications within 30 days	125 (17.9)	29 (28.4)	96 (16.1)	0.003
Length of stay in hospital after surgery, day	11.0 (10.4, 11.6)	14.0 (12.0, 16.0)	10.0 (9.4, 10.6)	0.001
All-cause 30-day mortality	5 (0.7)	2 (2.0)	3 (0.5)	0.156

Data are presented as number (%) or median (95% confidence interval)

*ICU* intensive care unit

### Association between anesthesia handover and delirium development

Apart from complete handover of anesthesia care, univariate analyses identified nine other factors that were associated with the risk of delirium development after surgery, including age, BMI, previous stroke, preoperative albumin < 30 g/L, intraoperative etomidate, ICU admission with intubation, prophylactic dexmedetomidine, postoperative propofol within 7 days, and pathologically diagnosed cancer (Online Resource 2). Of these, postoperative propofol within 7 days was not included in the multivariable model because it was related to ICU admission with intubation. After correction with the above confounding factors, complete handover of anesthesia care remained as an independent factor that was associated with an increased risk of postoperative delirium (OR 1.787, 95% CI 1.012–3.155, *P* = 0.046) (Table 4).

**Table 4:** Factors in association with postoperative delirium

Variable	Univariate analyses^a^		Multivariate analysis^b^	
	OR (95% CI)	*P* value	OR (95% CI)	*P* value
Complete handover of anesthesia care	1.687 (1.006–2.828)	0.041	1.787 (1.012–3.155)	0.046
Age, years	1.049 (1.019–1.081)	0.001	1.041 (1.008–1.074)	0.013
Body mass index, kg/m^2^	0.902 (0.853–0.954)	< 0.001	0.930 (0.878–0.985)	0.013
Previous stroke	1.692 (1.083–2.644)	0.021	1.785 (1.104–2.886)	0.018
Preoperative albumin < 30 g/L	2.473 (1.301–4.702)	0.006	1.700 (0.838–3.450)	0.142
Intraoperative use of etomidate	1.823 (1.212–2.742)	0.004	1.373 (0.879–2.143)	0.163
ICU admission with intubation	2.109 (1.365–3.257)	0.001	1.859 (1.162–2.974)	0.010
Prophylactic dexmedetomidine^c^	0.345 (0.222–0.537)	< 0.001	0.354 (0.223–0.560)	< 0.001
Postoperative propofol within 7 days^d^	1.570 (1.038–2.373)	0.032	–	–
Pathologically diagnosed cancer	0.519 (0.328–0.820)	0.005	0.610 (0.369–1.007)	0.053

^a^Postoperative delirium within 7 days was modeled as a function of a single factor in the univariate logistic regression analyses

^b^Postoperative delirium within 7 days was modeled as a function of all factors with a *P* value < 0.05 in the univariate analyses. Multivariate Logistic regression analysis was performed using a Enter procedure. Hosmer–Lemeshow test of goodness of fit of the model: *χ*^2^ = 7.651, *df* = 8, *P* = 0.468

^c^Administered as a continuous infusion at a rate of 0.1 µg/kg/h from ICU admission to 8 am of next morning [15]

^d^Not included in multivariable analysis because of correlation with ICU admission with intubation

## Discussion

Results of this analysis showed that in elderly patients who were admitted to the ICU after noncardiac surgery, intraoperative handover between anesthesia providers was associated with an increased risk of delirium development after correction for confounding factors.

The development of postoperative delirium results from the complex interaction of a variety of risk factors [1, 9, 20]. It is estimated that in 30–40% of cases, postoperative delirium is potentially preventable by reducing the exposure to known risk factors [21, 22]. In addition, some pharmacologic interventions, such as dexmedetomidine, melatonin and antipsychotics, have been investigated for preventing postoperative delirium [15, 23–27]. However, even with the effective prophylactic measures, delirium still occur in some patients [15]. Therefore, further efforts should be made to identify unrecognized risk factors.

With the increasing number and complexity of surgeries [10], handover of anesthesia care is inevitable in clinical practice. In the participating hospitals, day shift anesthesiologists worked from 8 am to 4 pm. Between 3 and 4 pm, patients of whom the surgeries were continuing were handed over to the next shift anesthesiologists. In addition, patients who underwent surgery beyond 10 pm were handed over to the night shift anesthesiologists. However, studies showed that current practice of intraoperative handover among anesthesia providers is not optimal [14]; and complete handover of intraoperative anesthesia care is associated with worse outcomes, including increased all-cause mortality and more major complications within 30 days after surgery [13, 14].

For the first time, our results showed that intraoperative handover of anesthesia care was associated with an increased risk of postoperative delirium. Reasons leading to this result may include the following. First, intraoperative handover of patients’ information might be insufficient. This was reported by previous studies [14]. Given that the incoming doctors did not completely understand patients’ condition, anesthetic management might be suboptimal during subsequent surgery and led to increased delirium. Second, at ICU admission, the handover of patients’ information might be further comprised between the successive anesthesiologists and the ICU physicians. This might have led to increased complications including delirium [28]. Third, patients with complete handover usually had their surgeries ended late and returned to the ward late. It was possible that this worsened patients’ sleep quality and increased delirium, as poor sleep quality is associated with increased delirium [29]. At last, patients required handover of anesthesia care usually underwent long-duration surgery, which might also increase delirium [30, 31]. However, the association between surgical duration and delirium development was not found in our results.

Our results also found that patients with complete handover had a higher incidence of non-delirium complications and stayed longer in hospital after surgery. These were in line with previously reported results [13]. On the other hand, a good handover of patients between anesthesiologists and intensive care unit physicians can be achieved using a protocol or checklist [32, 33], and is associated with improved outcomes including less unplanned extubation and shortened ventilation times [34]. However, whether good intraoperative handover among anesthesia providers can decrease delirium and improve outcomes has not been well investigated.

There are several limitations of this secondary analysis. First, in the original trial, only patients who were admitted to the ICU after surgery were recruited and half of them received prophylactic dexmedetomidine. These limited the generalisability of our results and might produce bias by reducing postoperative delirium. Second, patients with or without complete handover exhibited differences in some baseline and perioperative variables which might affect delirium development. Although a multivariate regression model was used to adjust for confounding factors, we cannot completely exclude the interference of these factors. Lastly, we cannot establish a causal relationship between handover of anesthesia care and development of postoperative delirium from a secondary analysis. However, our results provide clues for further interventional studies.

## Conclusions

Our results indicated that intraoperative handover of anesthesia care was associated with an increased risk of delirium development in elderly patients admitted to ICU after noncardiac surgery. Considering our results and others, studies investigating measures to improve intraoperative handover are urgently needed.

## Electronic supplementary material

Below is the link to the electronic supplementary material.

Supplementary material 1 (DOCX 29 KB)
