# Supplementary material for: Handover of anesthesia care is associated with an increased risk of delirium in elderly after major noncardiac surgery: results of a secondary analysis
Source: J Anesth. 2019 Feb 28;33(2):295–303. doi: 10.1007/s00540-019-02627-3 (PMC6443921; doi:10.1007/s00540-019-02627-3)
Supplement: Supplementary file 1 — Supplementary material 1 (DOCX 29 KB) [file 540_2019_2627_MOESM1_ESM.docx]

**Article title:** Handover of anesthesia care is associated with an increased risk of delirium in elderly after major noncardiac surgery: Results of a second analysis

**Author names:** Guang-Yu Liu^1^, Xian Su^1^, Zhao-Ting Meng^1^, Fan Cui^1^, Hong-Liang Li^2^, Sai-Nan Zhu^3^, Dong-Xin Wang^1*^

**Affiliation:** ^1^Department of Anesthesiology and Critical Care Medicine, Peking University First Hospital, Beijing, China; ^2^Department of Critical Care Medicine, Peking University Third Hospital, Beijing, China; ^3^Department of Biostatistics, Peking University First Hospital, Beijing, China.

**^*^Corresponding author:** Dong-Xin Wang, MD, PhD. Email: wangdongxin@hotmail.com

Online Resource 1. Definitions of postoperative complications

| Complications | Requirements for acceptance |
| --- | --- |
| Circulatory insufficiency | Requirement of inotropic agents or vasoconstrictors for more than 24 hours after surgery. |
| Acute myocardial infarction | Concentration of cardiac troponin I exceed the diagnostic criteria for myocardial infarction as well as new Q waves (lasts for 0.03 s) or continuous (4 days) abnormal ST‐T segment. |
| New onset arrhythmia | Confirmed by 12‐lead electrocardiogram and necessitated medical treatment and/or cardioversion. |
| Pulmonary infection | New infiltrate on chest radiograph combined with temperature over 38°C and leukocytosis. |
| Stroke | Persisted new focal neurologic deficit and confirmed by neurologic imaging. |
| Acute renal failure | New onset renal failure that required renal replacement therapy. |
| Wound dehiscence | Wound rupture that required secondary suturing. |
| Ileus | Lack of bowel movement, flatulence, and requirement of intravenous fluid therapy for more than one week after surgery. |
| Surgical bleeding | Bleeding after surgery that required secondary surgical hemostasis. |
| Anastomotic leakage | Extravasation of contrast agent in the body cavity or retroperitoneal space that required percutaneous drainage. |
| Gastrointestinal hemorrhage | Decrease of hemoglobin level combined with positive gastrointestinal occult blood test results that required treatment. |
| Wound infection | Pus expressed from the incision, and bacteria cultured from the pus. |
| Severe sepsis | Two or more criteria of systemic inflammatory response syndrome, with known infection and new onset dysfunction of at least one system. |
| Urinary tract infection | Confirmed by urinalysis and urine culture and necessitated antibiotic therapy. |

Online Resource 2. Factors in association with postoperative delirium (univariable analysis)

| Variables | N | OR (95% CI) | *P* value |
| --- | --- | --- | --- |
| Complete handover of anesthesia care | 102 | 1.687 (1.007-2.828) | 0.047 |
| Age, years | 700 | 1.049 (1.019-1.081) | 0.001 |
| Male gender | 423 | 1.489 (0.990-2.239) | 0.056 |
| Body mass index, kg/m^2^ | 700 | 0.902 (0.853-0.954) | <0.001 |
| Education, years | 700 | 1.007 (0.965-1.051) | 0.743 |
| Preoperative comorbidity |  |  |  |
| Previous stroke | 161 | 1.692 (1.083-2.644) | 0.021 |
| COPD | 42 | 1.487 (0.691-3.200) | 0.311 |
| Chronic smoking ^a^ | 176 | 1.320 (0.843-2.066) | 0.226 |
| Hypertension | 446 | 0.883 (0.582-1.340) | 0.558 |
| Coronary heart disease | 232 | 0.788 (0.505-1.229) | 0.293 |
| Diabetes mellitus | 190 | 0.840 (0.525-1.344) | 0.467 |
| Liver injury ^b^ | 19 | 1.937 (0.683-5.492) | 0.214 |
| Renal injury ^c^ | 35 | 1.911 (0.870-4.196) | 0.107 |
| Alcoholism ^d^ | 63 | 0.874 (0.418-1.826) | 0.721 |
| Previous surgery | 398 | 1.040 (0.690-1.567) | 0.853 |
| Chronic benzodiazepines | 61 | 1.044 (0.513-2.125) | 0.904 |
| Preoperative laboratory tests |  |  |  |
| Hematocrit <30% | 106 | 1.384 (0.816-2.345) | 0.228 |
| Albumin <30 g/L | 50 | 2.473 (1.301-4.702) | 0.006 |
| Glucose <4.0 or >10.0 mmol/L | 57 | 0.856 (0.394-1.861) | 0.695 |
| Na^+^ <135.0 or >145.0 mmol/L | 67 | 1.777 (0.973-3.245) | 0.062 |
| K^+^ <3.5 or >5.5 mmol/L | 73 | 1.049 (0.545-2.020) | 0.886 |
| ASA physical status class III | 302 | 1.482 (0.987-2.226) | 0.058 |
| Benzodiazepines at preoperative night | 79 | 1.051 (0.558-1.979) | 0.877 |
| Combined epidural-general anesthesia | 122 | 0.767 (0.434-1.357) | 0.363 |
| Intraoperative medication |  |  |  |
| Nitrous oxide | 523 | 0.765 (0.488-1.197) | 0.241 |
| Sevoflurane | 501 | 1.352 (0.842-2.171) | 0.213 |
| Benzodiazepines | 326 | 0.890 (0.592-1.339) | 0.576 |
| Propofol | 634 | 0.551 (0.301-1.007) | 0.053 |
| Etomidate | 273 | 1.823 (1.212-2.742) | 0.004 |
| Glucocorticoids | 646 | 0.716 (0.357-1.436) | 0.347 |
| Duration of anesthesia, hour | 700 | 1.063 (0.982-1.152) | 0.133 |
| Duration of surgery, hour | 700 | 1.060 (0.973-1.154) | 0.183 |
| Type of surgery |  |  |  |
| Superficial and transurethral | 69 | Ref. |  |
| Intra-abdominal | 475 | 0.683 (0.354-1.318) | 0.256 |
| Intra-thoracic | 120 | 1.011 (0.693-1.475) | 0.956 |
| Spinal and extremital | 36 | 1.183 (0.863-1.622) | 0.296 |
| Grade of surgery ^e^ |  |  |  |
| Intermediate | 42 | Ref. |  |
| Major or complex | 658 | 1.421 (0.546-3.699) | 0.472 |
| Cardiac risk of surgery ^f^ |  |  |  |
| Low | 33 | Ref. |  |
| Intermediate or high | 667 | 1.932 (0.579-6.444) | 0.284 |
| Estimated blood loss, 100 ml | 693 | 1.021 (0.997-1.047) | 0.092 |
| Total intraoperative fluid, 100ml | 700 | 1.008 (0.998-1.018) | 0.126 |
| Intraoperative blood transfusion | 114 | 1.530 (0.923-2.533) | 0.099 |
| ICU admission with intubation | 382 | 2.109 (1.365-3.257) | 0.001 |
| Prophylactic dexmedetomidine | 350 | 0.345 (0.222-0.537) | <0.001 |
| Postoperative analgesia |  |  |  |
| None | 73 | Ref. |  |
| PCIA | 516 | 1.003 (0.518-1.942) | 0.994 |
| PCEA | 111 | 0.857 (0.564-1.300) | 0.467 |
| Other sedatives/analgesics within 7 days |  |  |  |
| Propofol | 357 | 1.570 (1.038-2.373) | 0.032 |
| Benzodiazepines | 58 | 1.604 (0.834-3.083) | 0.157 |
| Opioids | 201 | 1.174 (0.757-1.820) | 0.475 |
| NSAIDs | 229 | 1.137 (0.743-1.742) | 0.554 |
| Pathologically diagnosed cancer | 561 | 0.519 (0.328-0.820) | 0.005 |

COPD, chronic obstructive pulmonary disease; PCIA, patient-controlled intravenous analgesia; PCEA, patient-controlled epidural analgesia; NSAIDS, non-steroid anti-inflammatory drugs.

^a^ Daily smoking of cigarettes up to half a pack for at least two years; ^b^ alanine aminotransferase and/or aspartate aminotransferase higher than 5 times of the normal upper limit; ^c^ serum creatinine level ≥177 μmol/L; ^d^ two drinks or more daily, or weekly consumption of the equivalent of 150mL of alcohol; ^e^ rated according to *NICE Guidance of Routine preoperative tests for elective surgery* [18]; ^f^ rated according to *ACC/AHA 2007 guidelines on perioperative cardiovascular evaluation and care for noncardiac surgery* [19].
